# Cross-population selection signatures in Canchim composite beef cattle

**DOI:** 10.1371/journal.pone.0264279

**Published:** 2022-04-01

**Authors:** Igor Nelson Herculano Duarte, Ayrton Fernandes de Oliveira Bessa, Luciana Diniz Rola, Maria Victória Henrique Genuíno, Iasmin Marques Rocha, Cintia Righetti Marcondes, Luciana Correia de Almeida Regitano, Danísio Prado Munari, Donagh Pearse Berry, Marcos Eli Buzanskas

**Affiliations:** 1 Departamento de Zootecnia, Universidade Federal da Paraíba, Areia, Paraíba, Brazil; 2 Embrapa Southeast Livestock, São Carlos, São Paulo, Brazil; 3 Departamento de Engenharia e Ciências Exatas, Universidade Estadual Paulista, Jaboticabal, São Paulo, Brazil; 4 Teagasc, Animal & Grassland Research and Innovation Centre, Moorepark, Fermoy Co. Cork., Ireland; USP FZEA: Universidade de Sao Paulo Faculdade de Zootecnia e Engenharia de Alimentos, BRAZIL

## Abstract

Analyses of livestock genomes have been used to detect selection signatures, which are genomic regions associated with traits under selection leading to a change in allele frequency. The objective of the present study was to characterize selection signatures in Canchim composite beef cattle using cross-population analyses with the founder Nelore and Charolais breeds. High-density single nucleotide polymorphism genotypes were available on 395 Canchim representing the target population, along with genotypes from 809 Nelore and 897 Charolais animals representing the reference populations. Most of the selection signatures were co-located with genes whose functions agree with the expectations of the breeding programs; these genes have previously been reported to associate with meat quality, as well as reproductive traits. Identified genes were related to immunity, adaptation, morphology, as well as behavior, could give new perspectives for understanding the genetic architecture of Canchim. Some selection signatures identified genes that were recently introduced in Canchim, such as the *loci* related to the polled trait.

## Introduction

In tropical countries, composite breed development generally attempts to combine the fitness traits of *Bos taurus indicus* with the reproductive and productive performance of *Bos taurus*. Canchim cattle from Brazil are a composite beef breed developed from crossbreeding, resulting in individuals with an approximate genetic composition of 62.50% Charolais and 37.50% Nelore [1].

Within the genome of populations subject to selection, distinct genetic patterns, called selection signatures, are regions with reduced genetic variability formed from selective pressure on a mutation over consecutive generations [2]. In-depth studies of such regions is now possible with the development of large-scale sequencing and genotyping platforms exploiting single nucleotide polymorphisms (SNP). Of the statistical methods developed to identify selection signatures, the cross-population extended haplotype homozygosity measures (XP-EHH and Rsb) detects alleles that are close to fixation or have actually achieved fixation in a given population, yet remaining polymorphic in the population as a whole [3, 4]; another method, the fixation index method (Fst), attempts to identify allele frequency differences between populations [5].

In Canchim, Urbinati et al. [6] used the extended haplotype homozygosity and the integrated haplotype score methods and observed selection signatures located on chromosomes 5 and 14 that were associated with pigmentation, productive, reproductive, and conformation traits. Cross-population methods have previously been used to identify divergent signals of selection signatures between Nelore cattle subpopulations from Brazil, in which three selection lines were evaluated and several candidate genes functionally related to growth metabolism were identified [7]. Selection signatures in the Charolais breed from Cuba and France have been reported to relate to adaptation [8]. Furthermore, Rodriguez-Valera et al. [8] identified genes related to immunity, metabolic changes, and heat tolerance in Charolais from Cuba within the detected selection signatures, while those related to muscle development and meat quality were described for French Charolais.

As Canchim originated from indicine beef cattle breeds (including Nelore), crossbred with Charolais, comparison of the genome of this breed with that of its founders can help identify genomic regions that have undergone recent selection. The objective therefore of the present study was to identify and characterize selection signatures in Canchim beef cattle using cross-population analyses including the Nelore and Charolais founder breeds.

## Materials and methods

### Genotype quality control and imputation

A total of 399 genotyped Canchim (CA) represented the target population while the reference populations (founder breeds) were composed of 814 Nelore (NE) and 897 Charolais (CH) purebred cattle. Canchim animals belonged to seven herds located in two Brazilian states (São Paulo and Goiás) and were raised in a pasture regime with mineral supplementation throughout the year, while Nelore animals were raised in individual or collective pens in feedlots located in the states of São Paulo and Mato Grosso do Sul. These states present tropical, warm, and rainy climate. Charolais animals were raised in Ireland, which has a temperate climate with the beef production relying heavily on *in situ* grazed perennial ryegrass pastures.

All animals were genotyped using the BovineHD BeadChip from Illumina which consists of 777,962 SNPs. Genotypes with GC scores lower than 0.55 were treated as missing. Genotype quality control was carried out using PLINK v.1.9 [9], in which SNPs and samples with a call rate lower than 90% and SNPs with minor allele frequency lower than 1% were excluded. The identity by state check revealed no unexpected correlations among samples. Only autosomal SNPs and SNPs with known positions based on the ARS-UCD1.2 bovine assembly [10] were retained.

The same quality control was applied to two datasets separately which differed just in the represented breeds: 1) Canchim and Nelore animals (CA vs. NE) and, 2) Canchim and Charolais (CA vs. CH). This approach aimed to avoid removing SNPs that were polymorphic in one breed but fixed in another. Following quality control, 693,531 SNPs remained in the joint CA vs. NE dataset with 707,626 SNPs remaining in CA vs. CH dataset. Sporadically missing genotypes were imputed using BEAGLE v.3.3.2 [11]. A total of 395 CA, 809 NE, and 897 CH animals met the quality control criteria. Principal component analysis, considering the three breeds in a single dataset with the same quality control parameters, was carried out by PLINK v.1.9 to evaluate population stratification.

### Selection signatures

Selection signatures were identified using the *rehh* package [12] from R [13]. The package uses the XP-EHH [4] and Rsb [3] methods. The PLINK v.1.9 software was used to obtain Fst values per SNP [14]. The XP-EHH and Rsb methods can identify haplotypes in high frequency by considering each SNP as a nucleus and comparing the integrated extended haplotype homozygosity (EHH) decay in each studied population [15]. Herein we set the limit value for the EHH decay at 0.05. Positive XP-EHH and Rsb values indicate selection signatures within the target population (i.e. CA). The standardized XP-EHH value for a Gaussian distribution, for a given SNP, was defined as:

$$\text{XP-EHH}=\frac{log(iEHH1/iEHH2)-\mu \;(iEHH1/iEHH2)}{\sigma (iEHH1/iEHH2)}$$ (1)

where *iEHH1* and *iEHH2* represent the integrated extended haplotype homozygosity of a central haplotype for the target (1) and reference (2) populations, respectively, *log* is the logarithm to the base 10, *μ* is the mean of *iEHH*1/*iEHH*2, and *σ* is the standard deviation of *iEHH*1/*iEHH*2. Similarly, the Rsb method compares EHH patterns using the median of the integrated extended haplotype homozygosity, instead of the mean, as defined for XP-EHH.

The Fst method is based on the difference in allele frequencies between populations and varies from zero to one; a higher value indicates large differences between populations. The Fst values were calculated as:

$$Fst=\frac{Ht-Hs}{Ht}$$ (2)

where *Ht* represents the total genetic heterozygosity for target and reference populations, and *Hs* represents the heterozygosity for the target population.

We considered using the 50 highest positive signal values for each of the three methods. According to each analysis (CA vs. NE and CA vs. CH), the SNPs identified in the XP-EHH, Rsb, and Fst were combined into two different files which were considered for functional analyses. Genes located within 250 kb of the center of the detected selection signature (SNP) were identified using the BIOMART tool from ENSEMBL [16]. Gene interactions were observed using the STRING website (https://string-db.org/).

## Results and discussion

**Fig 1:**
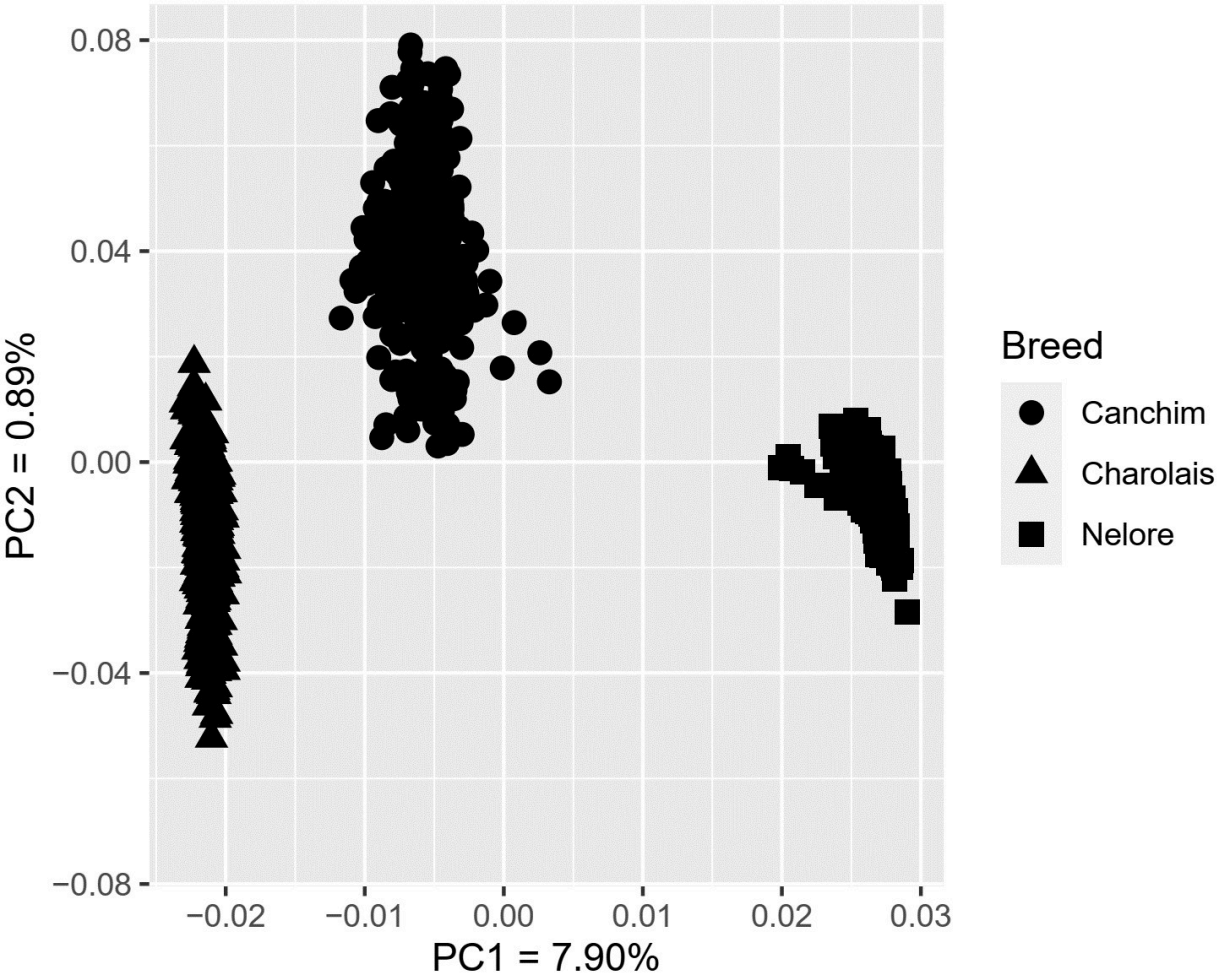
Plot of the first and second principal components (PC) for Canchim, Nelore, and Charolais breed.

Based on the principal component analysis, Canchim, Charolais, and Nelore clustered into three distinct groups (Fig 1), in which the first and second principal components explained 7.90% and 0.89%, respectively, of the total variance. Considering the genetic composition of Canchim, this result was consistent with our expectations since lower genetic distances were observed due to the greater contribution of Charolais in the Canchim. The mean Fst for CA vs. NE and CA vs. CH was equal to 0.24 and 0.10, respectively, in which low Fst values are related to low differentiation between breeds.

**Fig 2:**
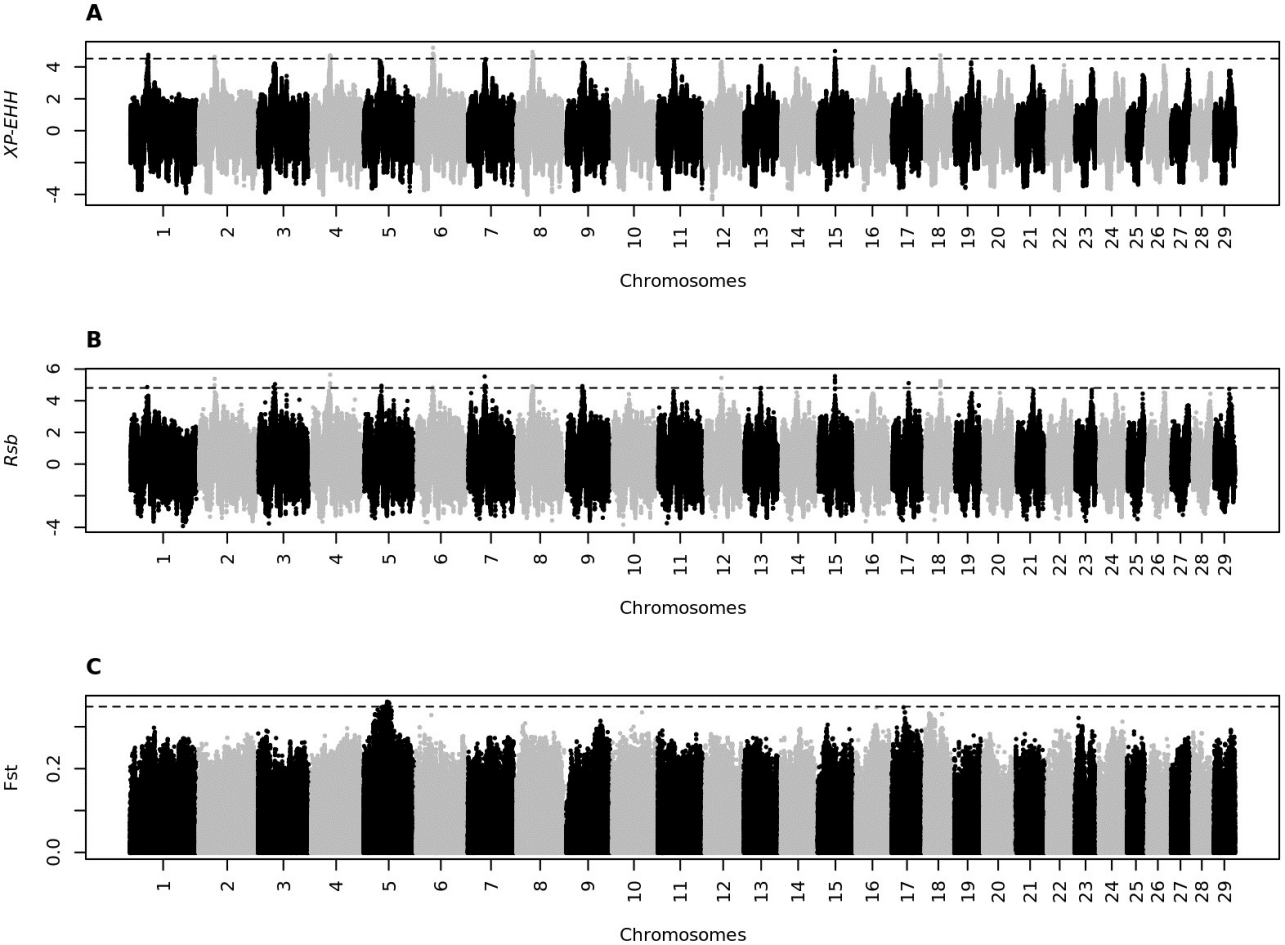
Manhattan plot for selection signature signals detected by the XP-EHH (A), Rsb (B), and Fst (C) methods for Canchim vs. Nelore.

**Fig 3:**
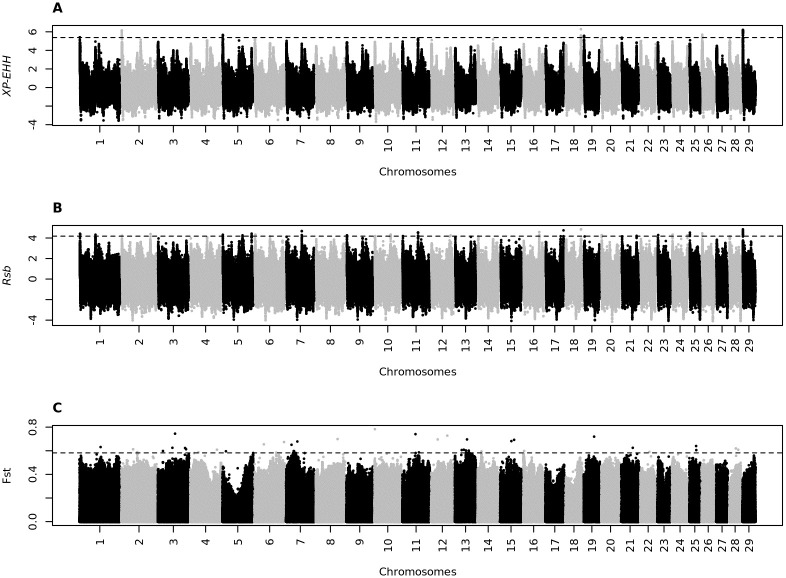
Manhattan plot for selection signature signals detected by the XP-EHH (A), Rsb (B), and Fst (C) methods for Canchim vs. Charolais.

Manhattan plots illustrating the selection signatures for CA vs. NE and CA vs. CH are in Figs 2 and 3, respectively. For the CA vs. NE analysis, a total of 325,675 and 332,279 SNPs demonstrated positive selection based on the XP-EHH and Rsb analysis, respectively; for the CA vs CH, the respective values were 322,525 and 342,234 SNPs.

When comparing CA vs. NE, only the GALNT18 and KCNIP4 genes were common to signatures detected using both the XP-EHH and Rsb methods (Table 1), whereas for CA vs. CH, only the FAT3 gene was common to both methods (Table 2). Selection signatures in common between Fst and the other methods were not observed. According to Evans et al. [17], each metric provides a distinct view of selection and that different selective forces are shaping these genomic regions.

**Table 1:** Genes located in selection signatures for Canchim vs. Nelore.

Methods	Chromosomes	Genes
**XP-EHH**	1	COL8A1
	4	LHFPL3
	6	KCNIP4
	8	UHRF2, bta-mir-2471, ENSBTAG00000052736
	10	MDGA2
	15	GALNT18
	18	ZFHX3
**Rsb**	1	EPHA6
	3	COL11A1
	4	LHFPL3
	5	PTPRR
	6	SLIT2, KCNIP4
	7	RACK1, TRIM41, SNORD96
	8	KDM4C, RCL1, SLC1A1, ENSBTAG00000052736
	9	FYN
	13	RALGAPA2, ENSBTAG00000048400
	15	GALNT18
	23	GMPR
	29	TMEM109
**Fst**	5	B4GALNT1, ENSBTAG00000049386, PIP4K2C, ENSBTAG00000051574, ENSBTAG00000051593, KIF5A, PIP4K2C, DCTN2, MBD6, ARHGAP9, GLI1, MARS, R3HDM2, STAC3, NXPH4, SHMT2, LRP1, MIP, TIMELESS, ORMDL2, SARNP, NTN4, ELK3, CDK17, CFAP54

**Table 2:** Genes located in selection signatures for Canchim vs. Charolais.

Methods	Chromosomes	Genes
**XP-EHH**	2	bta-mir-12062, CYFIP1
	18	LOC100139360
	26	LOC112444478
	29	FAT3
**Rsb**	1	IL10RB, URB1
	2	DOCK10
	5	TRHDE, DENND5B, PACSIN2
	7	ARHGEF37
	11	SNRNP200, LOC112448877, EHBP1
	12	SLC15A1
	20	DOCK2
	21	SYNE3
	23	DST
	24	CDH20
	25	PDPK1
	29	FAT3
**Fst**	1	VPS8
	2	DARS
	3	GOLPH3L, ZZZ3, CAP1, AGO3
	4	FAM126A, BRAF
	6	HERC6, ENSBTAG00000039647, TRMT44
	7	GNG7, CEP120, SNX24
	8	FGD3
	10	EPB41L4A
	13	MYO3A, ABHD12, FAM208B, SLC23A2, TCEA2, EEF1A2, KCNQ2, LAMA5
	14	NSMCE2
	15	ST5, ARRB1
	19	CDC6, WIPF2
	21	SRP54
	22	LRIG1
	25	C16orf92, DOC2A, FAM57B, ZNF629
	28	ZCCHC24

S1-S8 Tables in S1 File present the full description of SNPs, positions, and gene names for the detected selection signatures. No functions were identified for the ENSBTAG00000048400, ENSBTAG00000049386, ENSBTAG00000051574, ENSBTAG00000051593, ENSBTAG00000052736, TMEM109, UHRF2, bta-mir-12062, C16orf92, FAM126A, LOC100139360, LOC112444478, LOC112448877, and ZCCHC24 genes which were in the vicinity of the detected selection signatures. S1 and S2 Figs in S1 File present the gene interactions for CA vs. NE and CA vs. CH, respectively.

### CA vs. NE

Here and in the next sections, we discuss the genes co-located with the selection signatures. According to Liu et al. [18] and Flori et al. [19], the B4GALNT1 and CDK17 were candidate genes for adaptation to high altitude and the ability to adapt to the West African region, respectively. The SNORD96 gene was documented to reside within a selection signature associated with physiological adaptations against environmental stressors, such as resistance to infectious diseases, long drought periods, and food shortages [20]. The genes TIMELESS and ZFHX3 were indicated as essential for circadian rhythm and photo-entrainment [21, 22].

The EPHA6 and NTN4 genes were identified in a genome-wide association study for reactivity traits in Guzerat cattle, which is a phenotype based on the frequency and intensity of movements during the weighing in the chute [23]. These two genes were involved in biological processes related to the growth of axons in nerve cells and remodeling of neuronal projections during the development of the nervous system, respectively.

Using microsatellite markers, Gutiérrez-Gil et al. [24] reported that the DCTN2 gene was associated with coat color, being responsible for the dilution of eumelanin (black-brown pigment) and pheomelanin (red-yellow pigment). Other studies associated the SLC1A1, KIF5A, MIP, and TRIM41 genes with immune response [25], defense against parasites [26], degenerative spinal demyelination disease [27], respiratory diseases [28], and resistance to paratuberculosis infection [29], respectively, in cattle.

The selection signatures detected in the present study, which co-located with the ORMDL2 and SARNP genes, are in agreement with Zhao et al. [30], who identified these genes within selection signatures in the Simmental breed. In beef cattle, the GLI1, PIP4K2C, and RALGAPA2 genes were associated with tissue formation [31], lipid metabolism [32], and subcutaneous fat thickness [33], respectively. In pigs, the GMPR gene has been associated with intramuscular fat [34].

Both Leal-Gutiérrez et al. [35] and Caldas et al. [36] proposed CFAP54 and COL11A1 as candidate genes for meat tenderness and carcass quality. In Nelore cattle, Somavilla et al. [37] identified the COL8A1 gene, related to the multiplication of satellite cells and smooth muscle production, under a selection signature window. The ELK3, RCL1, PTPRR, and R3HDM2 genes have all been reported to be associated with feed conversion rate [38], daily weight gain [39], rib eye area [40], and meat quality [41] in beef cattle. The bta-mir-2471, MARS, and GALNT18 genes were documented to be associated with milk yield [42], milk protein percentage [43], and fat percentage [44] in the Holstein breed.

In Danish Red, Finnish Ayrshire, and Swedish Red cattle, Höglund et al. [45] reported that KCNIP4 and SLIT2 as associated with length in days of the interval from calving to first insemination and 56-day non-return rate, respectively. Walker and Biase [46] associated the RACK1 gene with the potential for higher development of oocytes. The NXPH4 and SHMT2 genes were associated with first calving interval in buffaloes [47]; while in pigs, the LHFPL3 gene was associated with number of piglets born alive [48]. The ARHGAP9 gene was shown to be differentially expressed in pre-implanted bovine embryos from cows supplemented with methionine, being associated with the modulation of gene expression in bovine blastocysts [49].

Sarakul et al. [50] reported associations of the FYN and NTN4 genes with semen volume, number of sperm, and sperm motility in dairy cattle from Thailand. In the Fleckvieh breed, Khayatzadeh et al. [51] observed an association between the MDGA2 gene with seminal volume in bulls. In goats, the KDM4C was highlighted as a candidate gene for spermatogenesis and male gamete generation [52].

### CA vs. CH

Srikanth et al. [53] and Taye et al. [54] identified that the DOCK10 and SLC23A2 genes were associated with thermotolerance in Holstein and African cattle. The FAM208B gene has been functionally characterized in mammals and is involved in adaptation to Arctic and Antarctic environments [55]. The URB1 and IL10RB genes have been identified as associated with the polled trait in indicine and taurine cattle [56, 57]; while the DST and ZZZ3 were related to pathways of horns development in cattle [58] and horn cancer in indicine cattle [59], respectively. The PDPK1 gene was identified as a selection signature for coat color in Brown Swiss, Jersey, and Norwegian Red cattle [60].

Neupane et al. [28] identified that the AGO3 and ARRB1 genes were associated with respiratory diseases in dairy and beef cattle. The HERC6, WIPF2, DENND5B, and PDPK1 genes were documented to be associated with antiviral response [61], disease resistance [42], genetic susceptibility to bovine tuberculosis (*Mycobacterium bovis*) [62], and tick resistance [63], respectively. The CDC6, PACSIN2, DOC2A, and SLC15A1 genes were linked to immune responses [64, 65], immune deficiency [66], and inflammation and antibacterial response [67]. In buffaloes, the GNG7 gene has been associated with behavioral traits linked to adaptation to stress and fear responses [68, 69]; while in sheep, the CEP120 gene has been reported as associated with cortisol response to stress [70].

The ARHGEF37, ARRB1, FGD3, and FGD3 genes were found as associated with residual feed intake [71], post-weaning weight gain [72], carcass weight [73], and dry matter intake [74], respectively. Genes related to fat thickness, fat deposition, and adipogenesis were found by Xu et al. [75] (EHBP1, CYFIP1, and EEF1A2), Mokry et al. [76] (DARS), Zhang et al. [77] (CDH20), Seong et al. [32] (EPB41L4A and TCEA2), Yamashita-Sugahara et al. [78] (FAM57B), Li et al. [79] (LAMA5), and Crespo-Piazuelo et al. [80] (ABHD12). In composite cattle, TRHDE and FAT3 genes were associated with meat palatability [81]; while in Nelore, the DOCK2 gene was associated with meat tenderness [82]. According to Tizioto et al. [83], the SNX24 gene was associated with iron mineral content in the *Longissimus dorsi* muscle of Nelore cattle.

Regarding reproductive traits, the SNRNP200, BRAF, and NSMCE2 genes were identified, respectively, for stayability [84], sexual precocity [85], and age at first calving [86] in Nelore cattle. The CYFIP1 and CAP1 genes were associated with calving interval [87] and preparation of the uterine environment for future pregnancy in cattle [88], respectively. The MYO3A gene has been associated with low fertility of bulls [89]. In pigs, the GOLPH3L gene was identified by Moe et al. [90] as associated with androsterone levels.

### Implications

Due to the contribution of the Nelore, which is a breed highly adapted to the tropical environment, to the Canchim, the identification of selection signatures harboring genes related to the capacity to adapt to climatic, health, and food adversities as well as genes associated with the immune system are in line with what is expected. Likewise, the selection signatures observed when comparing the Canchim and Charolais populations also identified genes with the same functions as in the other population comparison albeit they were not the same genes as observed for CA vs. NE. Therefore, Canchim has the benefits of both breeds, demonstrating the effect of complementarity.

As Canchim animals are classified by coat color, with light coat and cream color being especially sought, the observation in the present study of genes related to color dilution residing in selection signatures is not a surprise and corroborates with the Charolais breed standards. Genes related to fear response and cortisol response to stress were detected, which could be targeted for a better understanding of complex behavior traits and maintain welfare and safety of producers and animals.

Polledness was incorporated in the Canchim by using polled Charolais bulls from Argentina, United States of America, and England [91]. Despite being an easily distinguishable trait, the detection of genes associated with polledness in Canchim has been confirmed. In practical terms, the absence of horns has an economic impact by reducing bruising and injuries in the animals, facilitating feeding practices, and reducing the incidence of serious accidents with handlers [92].

Most of the detected selection signatures for CA vs. NE and CA vs. CH were identified in other studies that observed associations with production (body weight gains, fat deposition, carcass quality, and meat quality) and reproduction (age ate first calving, semen quality, heifer sexual precocity, and cows reproductive longevity) traits. Therefore, the results corroborate the focus of beef cattle breeding programs, especially for the Canchim, which is based on a selection index consisting on birth weight, weaning weight, yearling weight, scrotal circumference, and carcass merit at yearling [93].

We found a greater proportion of genes related to carcass quality and growth traits for CA vs. CH. We could assume that there was an effective introgression of these genes in Canchim due to a higher contribution of Charolais in the breed development, which has been observed by Buzanskas et al. [94] using admixture analyses. Furthermore, differences between selective pressures for Charolais and Nelore could also have contributed to the results herein observed.

Some selection signatures harbored genes whose functions are not yet known. These genes may play an important role in the characterization of Canchim cattle and should be evaluated in the future. Finally, the selection signatures harboring the GALNT18 and KCNIP4 (CA vs. NE) and FAT3 (CA vs. CH) genes, which were detected by both XP-EHH and Rsb methods, could be highlighted as reliable candidate selection signatures.

## Conclusion

The founder breeds were demonstrated to shape the genetic composition of the Canchim. Most of the selection signatures were co-located with genes whose functions agree with the expectations of the breeding programs; these genes have previously been reported to associate with weight gain and meat quality, as well as reproductive traits. Identified genes were related to immunity, adaptation, morphology, behavior, could give new perspectives for understanding the genetic architecture of Canchim. Some selection signatures identified genes that were recently introduced in Canchim, such as the *loci* related to the polled trait.

## Supporting information

S1 File (ZIP)
