# Supplementary material for: Cross-population selection signatures in Canchim composite beef cattle
Source: PLoS One. 2022 Apr 1;17(4):e0264279. doi: 10.1371/journal.pone.0264279 (PMC8975110; doi:10.1371/journal.pone.0264279)
Supplement: S1 File — (ZIP) [file pone.0264279.s001.zip › Supporting information.pdf]

S1 Table. Single nucleotide polymorphisms (SNPs) detected within selection signatures using the XP-EHH method for Canchim vs. Nelore.

SNPs	Chromosomes	Positions (bp)	Genes
rs43373882, rs43373876	1	43969653, 43970492	*COL8A1*
rs133724877, rs137378404, rs135245137	2	38282759, 38283463, 38286798	-
rs133674775, rs134644684, rs42471200, rs42471207, rs42471212, rs42471218, rs42471224, rs42471934, rs42471939, rs42471948, rs134892386	4	45697497, 45698369, 45699036, 45700086, 45701570, 45702050, 45702682, 45703187, 45703856, 45704599, 45705463	*LHFPL3*
rs716291922	6	38186904	-
rs137469616, rs132974874, rs43459708, rs43459713	6	40333619, 40336337, 40337219, 40338033	*KCNIP4*
rs109104073, rs135117154, rs136470098, rs109818216, rs135612728	6	42137079, 42137637, 42138188, 42140051, 42140911	-
rs135738839, rs136767379	7	42432559, 42433284	-
rs132938393, rs137119854, rs135616277	8	38328447, 38331262, 38332234	*UHRF2*
rs136270294, rs110627495, rs41593266, rs134621033, rs109259605, rs133901412, rs134923103, rs109241941, rs134795758, rs134182629, rs133500601	8	40314619, 40315227, 40316507, 40317171, 40317748, 40318896, 40319771, 40329631, 40330350, 40335161, 40336502	*bta-mir-2471, ENSBTAG00000052736*
rs135276636	10	40326781	*MDGA2*
rs42840819, rs109834160, rs42840830, rs42841850, rs42841861	15	41366558, 41367107, 41367762, 41369314, 41369768	*GALNT18*
rs41876684, rs109862704	18	37901390, 38264595	*ZFHX3*

bp = base pairs.

S2 Table. Single nucleotide polymorphisms (SNPs) detected within selection signatures using the Rsb method for Canchim vs. Nelore.

SNPs	Chromosomes	Positions (bp)	Genes
rs134894939	1	41449949	*EPHA6*
rs132823008, rs137065990	2	38287973, 38288717	-
rs109115790, rs42698728	3	36849423, 40457717	*COL11A1*
rs42471954, rs42471170, rs134197904	4	45691887, 45693148, 45695444	*LHFPL3*
rs136234289, rs132666336	5	42843848, 42847905	*PTPRR*
rs110254965, rs110582067	6	39900932, 40810991	*SLIT2, KCNIP4*
rs135733085	7	40354752	-
rs109511991, rs109885568, rs136931967	7	40360157, 40366289, 40375392	*RACK1, TRIM41, SNORD96*
rs137551678, rs109233282	7	42434579, 42437700	-
rs137062772, rs109701770	8	37894518, 39722951	*KDM4C, RCL1*
rs109430733, rs135624744	8	39989437, 39994088,	*SLC1A1*
rs134621033	8	40317171	*ENSBTAG00000052736*
rs133655004, rs110514237, rs111010424, rs109032596, rs109588029, rs110431772	9	38702778, 38706607, 38708576, 38711154, 38714703, 38717048	*FYN*
rs110595901, rs730762781, rs109618046	12	39392471, 39392471, 39402681	-
rs136767326, rs108966909	13	40164552, 41434789	*RALGAPA2, ENSBTAG00000048400*
rs134487132, rs110478043, rs135402182, rs42840797, rs42840812, rs109834160, rs42840830	15	41359654, 41364340, 41364795, 41365267, 41365975, 41367107, 41367762	*GALNT18*
rs42309118	17	40512921	-
rs110346890, rs134244140, rs136604325, rs29027483, rs110710880	18	37892870, 37897576, 37899251, 37902039, 37902822	-
rs41984911	21	42702695	-
rs136111788	23	40866620	*GMPR*
rs109453088	29	37256700	*TMEM109*

bp = base pairs.

S3 Table. Single nucleotide polymorphisms (SNPs) detected within selection signatures using the Fst method for Canchim vs. Nelore.

SNPs	Chromosomes	Positions (bp)	Genes
rs133648303, rs135102974, rs136665949	5	55849254, 55853705, 55858809	*B4GALNT1, ENSBTAG00000049386*
rs134702544, rs132791408	5	55891486, 55895119	*PIP4K2C*
rs134673457, rs136047436, rs136966408, rs135353977, rs132630546, rs135775229	5	55903197, 55918952, 55930200, 55935707, 55940985, 55946164	*ENSBTAG00000051574, ENSBTAG00000051593, KIF5A, PIP4K2C, DCTN2, MBD6*
rs136819244, rs134826942, rs137705320	5	55989955, 55992815, 55999321	*ARHGAP9, GLI1, MARS*
rs136968991	5	56027323	-
rs136549932, rs134141555, rs135494743, rs132912942, rs137282168, rs135870984, rs136863768, rs135049400, rs137017835, rs135015894, rs132796363, rs136553412, rs134078306, rs137606680	5	56042169, 56050586, 56058232, 56063120, 56089779, 56096333, 56100012, 56108889, 56144768, 56148237, 56154876, 56169322, 56172984, 56178350	*R3HDM2*
rs133424563	5	56190088	*STAC3*
rs136245785	5	56216047	*NXPH4, SHMT2*
rs135718080	5	56238625	*LRP1*
rs17871459	5	56935291	*MIP, TIMELESS*
rs135419986	5	57452342	*ORMDL2, SARNP*
rs136121620	5	59647243	-
rs137435186	5	59806385	-
rs133792373	5	60042709	*NTN4*
rs134094575	5	60394885	-
rs135099773	5	60471598	-
rs136634837, rs134840653, rs133479662	5	60475965, 60517054, 60532509	*ELK3*
rs133612722, rs135042890, rs133287150, rs135954955	5	60556462, 60559059, 60593993, 60660156	*CDK17*
rs134395999	5	60711581	-
rs134499135, rs378652793, rs380984130	5	60820517, 60820414, 60820436	*CFAP54*

bp = base pairs.

S4 Table. Single nucleotide polymorphisms (SNPs) detected within selection signatures using the XP-EHH method for Canchim vs. Charolais.

SNPs	Chromosomes	Positions (bp)	Genes
rs110087191, rs110110458	1	1585123, 1585904	-
rs110691471, rs43596208, rs43596193, rs109179645, rs43596186, rs43596167, rs43596156, rs43596153, rs43596141	2	1095926, 1097698, 1099026, 1100377, 1102601, 1105385, 1106317, 1106829, 1108259	*bta-mir-12062, CYFIP1*
rs41571559, rs132986546, rs42741672, rs135525239, rs136845218	5	1900517, 1901477, 1903592, 1905484, 1906775	-
rs41895000, rs41895010	18	59992350, 59996532	-
rs41894946, rs109975245	18	60011054, 60011920	*LOC100139360*
rs110624318, rs133214613, rs134688057, rs110707315, rs110849836	19	1451084, 1464314, 1466209, 1467093, 1468294	-
rs110183337	26	2782291	*LOC112444478*
rs110399112, rs132707265, rs109441882, rs110430476, rs110829774, rs135082591, rs136111956	26	2783512, 2784434, 2789785, 2791182, 2793255, 2795689, 2797025	-
rs42155898, rs108942481, rs42155904, rs42155913, rs42155919, rs42155927, rs42156438, rs42156449, rs42156467, rs42156482, rs42156490, rs42156506, rs42156521, rs42156527, rs110636081, rs42157241, rs42951138	29	2348783, 2349935, 2350580, 2351334, 2351928, 2352983, 2354443, 2355314, 2357283, 2359313, 2360091, 2361558, 2362208, 2362913, 2364158, 2364986, 2373405	*FAT3*

bp = base pairs.

S5 Table. Single nucleotide polymorphisms (SNPs) detected within selection signatures using the Rsb method for Canchim vs. Charolais.

SNPs	Chromosomes	Positions (bp)	Genes
rs110969048, rs109874675, rs42624482	1	2277019, 3168711, 62490666	*IL10RB, URB1*
rs43284969, rs43319784	2	1654678, 112895681	*DOCK10*
rs132639698, rs42661975	5	1949473, 2050090	*TRHDE*
rs109830520	5	78518915	*DENND5B*
rs132978041, rs133514074	5	113790790, 113798066	*PACSIN2*
rs43447008	6	3489757	-
rs137321540, rs110824104, rs110266195	7	60976830, 60977404, 60981556	*ARHGEF37*
rs135873851	9	2749095	-
rs109158474, rs134445404, rs133518196, rs134774043	10	62862939, 62885833, 62887972, 62954022	-
rs43656766	11	2396722	*SNRNP200*
rs41618429, rs136742616	11	61126116, 61127176	-
rs110151243, rs109671580	11	61137864, 61176563	*LOC112448877, EHBP1*
rs110191319	12	75664218	*SLC15A1*
rs110566736, rs136962946, rs109689439	16	62299327, 62299977, 62303238	-
rs137132272	17	70487748	-
rs137226482, rs42646460	18	60014326, 60880768	-
rs110229380, rs109849813	20	1684923, 1693617	*DOCK2*
rs137018202	21	60291728	*SYNE3*
rs136110841	23	3705093	*DST*
rs43067620, rs134048411	24	1895917, 60091073	*CDH20*
rs110135987, rs134453491, rs109253760	25	2078993, 2080676, 2086193	*PDPK1*
rs132707265, rs135088369	26	2784434, 2822109	-
rs42155919, rs42156482, rs110189006, rs110657088, rs43083340, rs43083332, rs43082920, rs43082915	29	2351928, 2359313, 2371374, 2374000, 2432821, 2433400, 2437018, 2438397	*FAT3*

bp = base pairs.

S6 Table. Single nucleotide polymorphisms (SNPs) detected within selection signatures using the Fst method for Canchim vs. Charolais.

SNPs	Chromosomes	Positions (bp)	Genes
rs133369024	1	82308847	*VPS8*
rs134091143, rs134090983	2	45236595, 61422465	*DARS*
rs136155615, rs135406956, rs133454274, rs109355328, rs133358863	3	20042268, 56724756, 67033710, 106050380, 109834712	*GOLPH3L, ZZZ3, CAP1, AGO3*
rs136432956, rs136674703	4	31618793, 104050494	*FAM126A, BRAF*
rs135552378	5	13980342	-
rs110335332, rs137598038, rs135729225	6	36334803, 84700812, 114723327	*HERC6, ENSBTAG00000039647, TRMT44*
rs133797808, rs132646622, rs134104203, rs110415002, rs134884972, rs133407105, rs134090635, rs110014023	7	21041110, 28200770, 29651126, 29690893, 30006314, 30367487, 30813812, 43772010	*GNG7, CEP120, SNX24*
rs137779301	8	84398111	*FGD3*
rs136226658	10	1654843	*EPB41L4A*
rs133449931, rs110582254	11	51137373, 64484316	-
rs133903827, rs134114175	12	25589790, 62818654	-
rs135644451, rs137119077, rs134372017, rs137431719, rs42781378, rs135843840, rs133677379, rs132698220, rs135693681, rs135913428, rs136475322	13	26615101, 34839367, 42839858, 42997272, 47339906, 53811244, 54160112, 54178836, 54205372, 54771089, 54889778	*MYO3A, ABHD12, FAM208B, SLC23A2, TCEA2, EEF1A2, KCNQ2, LAMA5*
rs133024734	14	15021635	*NSMCE2*
rs133280318, rs135187499	15	43817350, 54510608	*ST5, ARRB1*
rs134950764	16	3569787	-
rs136952600	19	40525155	*CDC6, WIPF2*
rs136332953	21	45315228	*SRP54*
rs134724349	22	35054886	*LRIG1*
rs135683791, rs42069061	25	26250078, 26904680	*C16orf92, DOC2A, FAM57B, ZNF629*
rs134840402, rs135960665	28	25924549, 35053844	*ZCCHC24*

bp = base pairs.

S7 Table. Genes located within the selection signatures for Canchim vs. Nelore.

Ensembl ID	Gene ID	Gene Names
ENSBTAG00000018403	ARHGAP9	Rho GTPase activating protein 9
ENSBTAG00000004494	B4GALNT1	Bos taurus beta-1,4-N-acetyl-galactosaminyltransferase 1 (B4GALNT1), mRNA.
ENSBTAG00000045466	bta-mir-2471	bta-mir-2471
ENSBTAG00000001510	CDK17	cyclin dependent kinase 17
ENSBTAG00000015792	CFAP54	cilia and flagella associated protein 54
ENSBTAG00000021217	COL11A1	Bos taurus collagen type XI alpha 1 chain (COL11A1), mRNA.
ENSBTAG00000013662	COL8A1	Bos taurus collagen type VIII alpha 1 chain (COL8A1), mRNA.
ENSBTAG00000010624	DCTN2	dynactin subunit 2
ENSBTAG00000001509	ELK3	Bos taurus ELK3, ETS transcription factor (ELK3), mRNA.
ENSBTAG00000044111	EPHA6	EPH receptor A6
ENSBTAG00000011851	FYN	Bos taurus FYN proto-oncogene, Src family tyrosine kinase (FYN), mRNA.
ENSBTAG00000002914	GALNT18	polypeptide N-acetylgalactosaminyltransferase 18
ENSBTAG00000006631	GLI1	Bos taurus GLI family zinc finger 1 (GLI1), mRNA.
ENSBTAG00000015743	GMPR	guanosine monophosphate reductase
ENSBTAG00000047743	KCNIP4	Bos taurus potassium voltage-gated channel interacting protein 4 (KCNIP4), mRNA.
ENSBTAG00000043987	KDM4C	lysine demethylase 4C
ENSBTAG00000021336	KIF5A	kinesin family member 5A
ENSBTAG00000048818	LHFPL3	LHFPL tetraspan subfamily member 3
ENSBTAG00000010830	LRP1	LDL receptor related protein 1
ENSBTAG00000018405	MARS	methionyl-tRNA synthetase
ENSBTAG00000010616	MBD6	methyl-CpG binding domain protein 6
ENSBTAG00000005697	MDGA2	MAM domain containing glycosylphosphatidylinositol anchor 2
ENSBTAG00000010127	MIP	major intrinsic protein of lens fiber
ENSBTAG00000003183	NTN4	Bos taurus netrin 4 (NTN4), mRNA.
ENSBTAG00000047650	NXPH4	neurexophilin 4
ENSBTAG00000020663	ORMDL2	Bos taurus ORMDL sphingolipid biosynthesis regulator 2 (ORMDL2), mRNA.
ENSBTAG00000003942	PIP4K2C	Bos taurus phosphatidylinositol-5-phosphate 4-kinase type 2 gamma (PIP4K2C), mRNA.
ENSBTAG00000015311	PTPRR	Bos taurus protein tyrosine phosphatase, receptor type R (PTPRR), transcript variant 2, mRNA.
ENSBTAG00000018361	R3HDM2	Bos taurus R3H domain containing 2 (R3HDM2), mRNA.
ENSBTAG00000019648	RACK1	receptor for activated C kinase 1
ENSBTAG00000014178	RALGAPA2	Ral GTPase activating protein catalytic alpha subunit 2
ENSBTAG00000018667	RCL1	Bos taurus RNA terminal phosphate cyclase like 1 (RCL1), mRNA.
ENSBTAG00000020662	SARNP	Bos taurus SAP domain containing ribonucleoprotein (SARNP), mRNA.
ENSBTAG00000031500	SHMT2	Bos taurus serine hydroxymethyltransferase 2 (SHMT2), mRNA.
ENSBTAG00000019125	SLC1A1	solute carrier family 1 member 1
ENSBTAG00000005108	SLIT2	slit guidance ligand 2
ENSBTAG00000042936	SNORD96	Small nucleolar RNA SNORD96 family
ENSBTAG00000018358	STAC3	SH3 and cysteine rich domain 3
ENSBTAG00000020459	TIMELESS	Bos taurus timeless circadian regulator (TIMELESS), mRNA.
ENSBTAG00000018362	TMEM109	Bos taurus transmembrane protein 109 (TMEM109), mRNA.
ENSBTAG00000019646	TRIM41	Bos taurus tripartite motif containing 41 (TRIM41), mRNA.
ENSBTAG00000020815	UHRF2	ubiquitin like with PHD and ring finger domains 2
ENSBTAG00000014636	ZFHX3	zinc finger homeobox 3
ENSBTAG00000048400	-	-
ENSBTAG00000049386	-	-
ENSBTAG00000051574	-	-
ENSBTAG00000051593	-	-
ENSBTAG00000052736	-	-

S8 Table. Genes located within the selection signatures for Canchim vs. Charolais.

Ensembl ID	Gene ID	Gene Names
ENSBTAG00000001420	ABHD12	Bos taurus abhydrolase domain containing 12 (ABHD12), mRNA.
ENSBTAG00000018324	AGO3	Bos taurus argonaute RISC catalytic component 3 (AGO3), mRNA.
ENSBTAG00000015419	ARHGEF37	Bos taurus Rho guanine nucleotide exchange factor (GEF) 37 (ARHGEF37), mRNA.
ENSBTAG00000020485	ARRB1	arrestin beta 1
ENSBTAG00000021761	BRAF	B-Raf proto-oncogene, serine/threonine kinase
ENSBTAG00000052889	bta-mir-12062	bta-mir-12062
ENSBTAG00000048797	C16orf92	chromosome 16 open reading frame 92
ENSBTAG00000013363	CAP1	cyclase associated actin cytoskeleton regulatory protein 1
ENSBTAG00000010384	CDC6	cell division cycle 6
ENSBTAG00000001739	CDH20	cadherin 20
ENSBTAG00000013184	CEP120	Bos taurus centrosomal protein 120 (CEP120), mRNA.
ENSBTAG00000016894	CYFIP1	Bos taurus cytoplasmic FMR1 interacting protein 1 (CYFIP1), mRNA.
ENSBTAG00000009949	DARS	aspartyl-tRNA synthetase
ENSBTAG00000020315	DENND5B	Bos taurus DENN domain containing 5B (DENND5B), mRNA.
ENSBTAG00000005031	DOC2A	Bos taurus double C2 domain alpha (DOC2A), mRNA.
ENSBTAG00000000581	DOCK10	Bos taurus dedicator of cytokinesis 10 (DOCK10), mRNA.
ENSBTAG00000014612	DOCK2	dedicator of cytokinesis 2
ENSBTAG00000021237	DST	dystonin
ENSBTAG00000021685	EEF1A2	eukaryotic translation elongation factor 1 alpha 2
ENSBTAG00000044173	EHBP1	EH domain binding protein 1
ENSBTAG00000014750	EPB41L4A	Bos taurus erythrocyte membrane protein band 4.1 like 4A (EPB41L4A), mRNA.
ENSBTAG00000004575	FAM126A	family with sequence similarity 126 member A
ENSBTAG00000019197	FAM208B	family with sequence similarity 208 member B
ENSBTAG00000005033	FAM57B	Bos taurus family with sequence similarity 57 member B (FAM57B), mRNA.
ENSBTAG00000004081	FAT3	Bos taurus FAT atypical cadherin 3 (FAT3), mRNA.
ENSBTAG00000006939	FGD3	Bos taurus FYVE, RhoGEF and PH domain containing 3 (FGD3), mRNA.
ENSBTAG00000007644	GNG7	Bos taurus G protein subunit gamma 7 (GNG7), mRNA.
ENSBTAG00000020357	GOLPH3L	golgi phosphoprotein 3 like
ENSBTAG00000020536	HERC6	Bos taurus HECT and RLD domain containing E3 ubiquitin protein ligase family member 6 (HERC6), mRNA.
ENSBTAG00000019404	IL10RB	Bos taurus interleukin 10 receptor subunit beta (IL10RB), mRNA.
ENSBTAG00000003030	KCNQ2	potassium voltage-gated channel subfamily Q member 2
ENSBTAG00000003061	LAMA5	laminin subunit alpha 5
ENSBTAG00000010360	LRIG1	leucine rich repeats and immunoglobulin like domains 1
ENSBTAG00000002123	MYO3A	myosin IIIA
ENSBTAG00000009394	NSMCE2	NSE2 (MMS21) homolog, SMC5-SMC6 complex SUMO ligase
ENSBTAG00000019290	PACSIN2	Bos taurus protein kinase C and casein kinase substrate in neurons 2 (PACSIN2), mRNA.
ENSBTAG00000034436	PDPK1	3-phosphoinositide dependent protein kinase 1
ENSBTAG00000004440	SLC15A1	Bos taurus solute carrier family 15 member 1 (SLC15A1), mRNA.
ENSBTAG00000032366	SLC23A2	Bos taurus solute carrier family 23 member 2 (SLC23A2), mRNA.
ENSBTAG00000000848	SNRNP200	Bos taurus small nuclear ribonucleoprotein U5 subunit 200 (SNRNP200), mRNA.
ENSBTAG00000031069	SNX24	Bos taurus sorting nexin 24 (SNX24), mRNA.
ENSBTAG00000019085	SRP54	Bos taurus signal recognition particle 54 (SRP54), mRNA.
ENSBTAG00000005356	ST5	suppression of tumorigenicity 5
ENSBTAG00000003526	SYNE3	Bos taurus spectrin repeat containing nuclear envelope family member 3 (SYNE3), mRNA.
ENSBTAG00000021836	TCEA2	transcription elongation factor A2
ENSBTAG00000018575	TRHDE	thyrotropin releasing hormone degrading enzyme
ENSBTAG00000004797	TRMT44	tRNA methyltransferase 44 homolog
ENSBTAG00000012412	URB1	Bos taurus URB1 ribosome biogenesis 1 homolog (S. cerevisiae) (URB1), mRNA.
ENSBTAG00000005209	VPS8	VPS8, CORVET complex subunit
ENSBTAG00000038126	WIPF2	Bos taurus WAS/WASL interacting protein family member 2 (WIPF2), mRNA.
ENSBTAG00000003877	ZCCHC24	zinc finger CCHC-type containing 24
ENSBTAG00000026307	ZNF629	Bos taurus zinc finger protein 629 (ZNF629), mRNA.
ENSBTAG00000017419	ZZZ3	Bos taurus zinc finger ZZ-type containing 3 (ZZZ3), mRNA.
-	LOC100139360	-
-	LOC112444478	-
-	LOC112448877	-
