# Supplementary figures and images for: Cross-population selection signatures in Canchim composite beef cattle
Source: PLoS One. 2022 Apr 1;17(4):e0264279. doi: 10.1371/journal.pone.0264279 (PMC8975110; doi:10.1371/journal.pone.0264279)

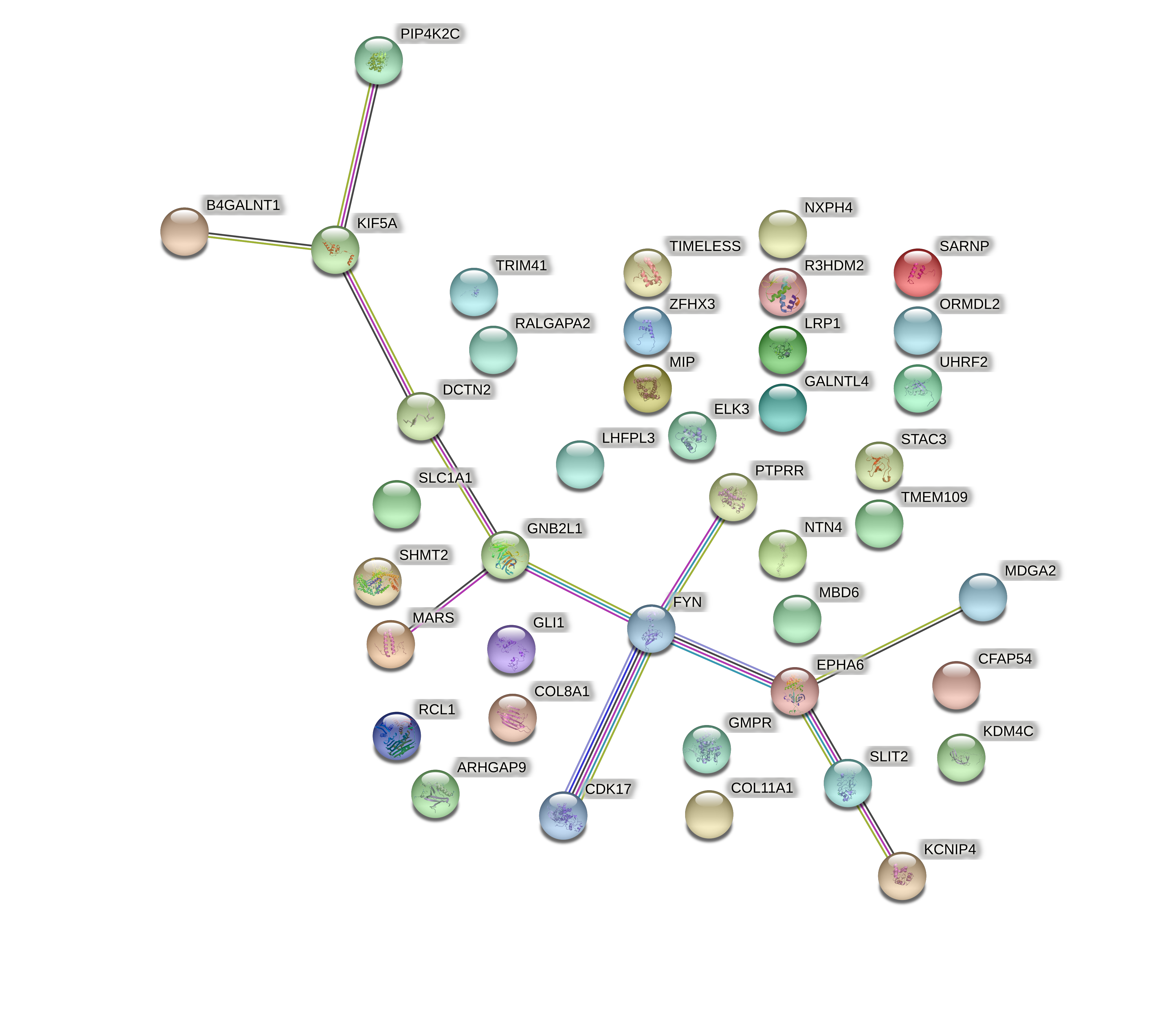

Supplement: S1 File — (ZIP) [file pone.0264279.s001.zip › Fig S1.tiff]

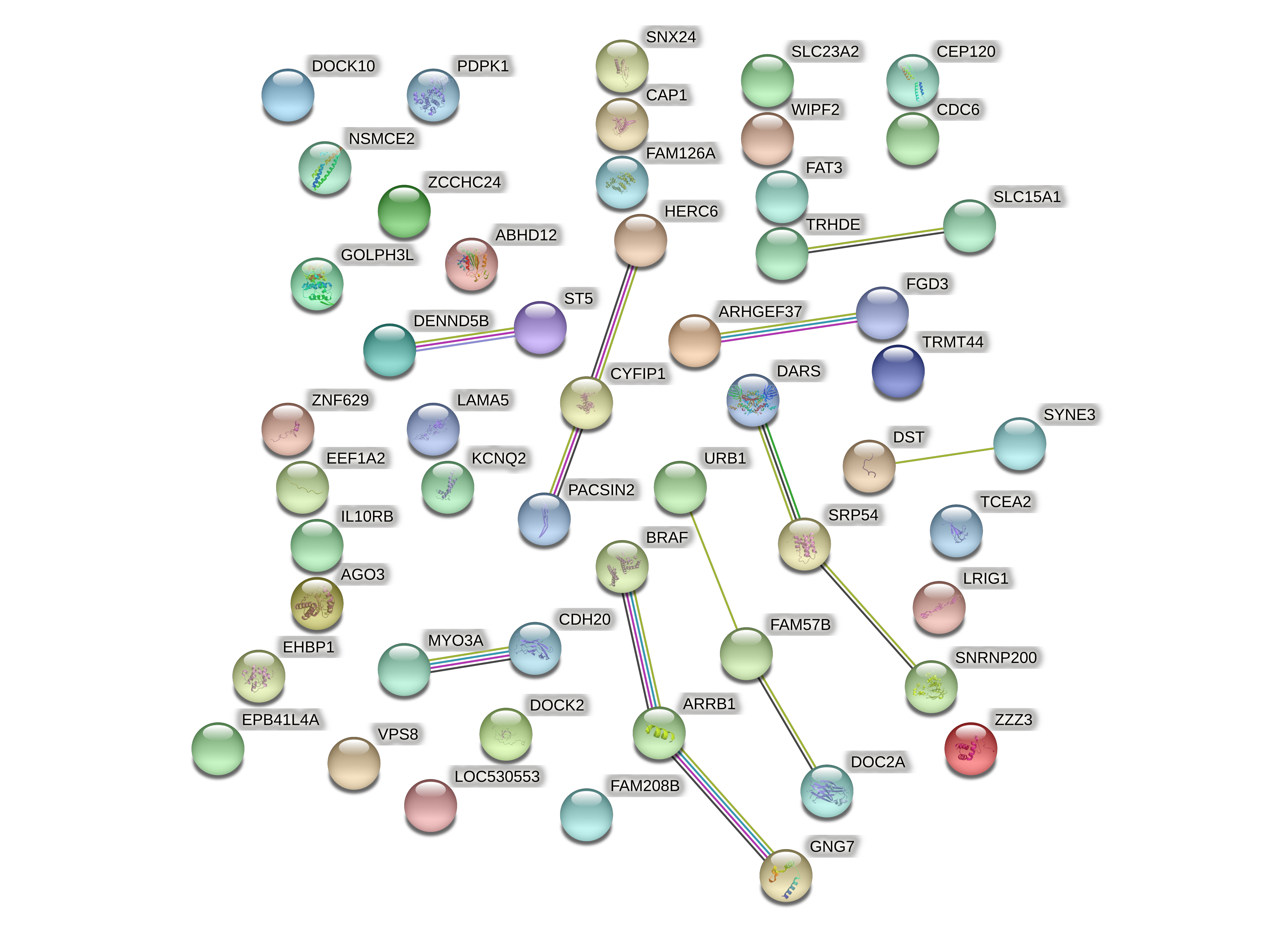

Supplement: S1 File — (ZIP) [file pone.0264279.s001.zip › Fig S2.tiff]
